# Hogweed Seed Oil: Physico–Chemical Characterization, LC-MS Profile, and Neuroprotective Activity of *Heracleum dissectum* Nanosuspension

**DOI:** 10.3390/life13051112

**Published:** 2023-04-29

**Authors:** Daniil N. Olennikov, Nadezhda K. Chirikova

**Affiliations:** 1 Laboratory of Medical and Biological Research, Institute of General and Experimental Biology, Siberian Division, Russian Academy of Science, 6 Sakhyanovoy Street, 670047 Ulan-Ude, Russia; 2 Department of Biochemistry and Biotechnology, North-Eastern Federal University, 58 Belinsky Street, 677027 Yakutsk, Russia; hofnung@mail.ru

**Keywords:** hogweed oil, coumarins, mass spectrometry, quantification, storage stability, CO_2_-assisted effervescence, brain ischemia

## Abstract

The seeds of dissected hogweed (*Heracleum dissectum* Ledeb., Apiaceae) are the source of hogweed oil (HSO), which is still underexplored and requires careful chemical and biological studies. The performed physico–chemical analysis of HSO elucidated basic physical characteristics and revealed the presence of fatty acids, essential oil components, pigments, and coumarins. High-performance liquid chromatography with photodiode array detection and electrospray ionization triple quadrupole mass spectrometric detection (HPLC–PDA–ESI–tQ–MS/MS) identified 38 coumarins that were characterized and quantified. Various furanocoumarins were the major components of HSO polyphenolics, including imperatorin, phellopterin, and isoimperatorin, and the total coumarin content in HSO varied from 181.14 to 238.42 mg/mL. The analysis of storage stability of the selected compounds in HSO indicated their good preservation after 3-year storage at cold and freezing temperatures. The application of the CO_2_-assisted effervescence method allowed the production of an HSO nanosuspension, which was used in a brain ischemia model of rats. The HSO nanosuspension enhanced cerebral hemodynamics and decreased the frequency of necrotic processes in the brain tissue. Thus, *H. dissectum* seeds are a good source of coumarins, and HSO nanosuspension promotes neuroprotection of the brain after lesions, which supports earlier ethnopharmacological data.

## 1. Introduction

The Apiaceae family, which contains 446 accepted genera and approximately 4000 species, is a source of useful plants that grow throughout the world [1]. The members of the Apiaceae family are valuable crops with nutraceutical significance [2] and bioactive medicinal plants [3]. Asia has the greatest variety of apiaceous species numbering approximately 300 species [4], including a widely distributed genus *Heracleum* (hogweed), which counts 90 species [5]. Various hogweeds have a long history of use by humans as medicinal and food plants [6]. Dissected hogweed (*Heracleum dissectum* Ledeb.) is a large Asian plant; it grows in coniferous, coniferous-broad-leaved, and broad-leaved valley and mountain large-grass forests, thickets of shrubs, on the edges, glades, and tall grass meadows of Western, Middle, and Eastern Siberia, the Far East of Russia, Kyrgyzstan, Kazakhstan, Mongolia, China, Korea, and Japan [7].

Dissected hogweed is a perennial polycarpic plant that is 70–160 cm in height (up to 2 m). Caudexes are unbranched, and taproots are branching. Stems are solitary, hollow, deeply furrowed, protruding pubescent, and corymbose branching in upper parts. Basal leaves are on long petioles. Petioles of basal leaves are hollow or dense, and leaf blades are 20–50 cm long and 15–35 cm in size, trifoliate, less often sessile, deeply palmate-lobed, and serrated along the edge. Central umbels are 13–25 cm in diameter, with 20–40 protruding, softly pubescent rays up to 10 cm long. The petals of the flower are white; the outer petals of the marginal flowers are greatly enlarged, up to 10 mm elongated. Seeds (fruits) are 6–16 mm long, ovoid, or obovate in outline. Carpophore is bifid. Mericarps are compressed dorsally, rounded oval or ovate, glabrous, or covered with sparse hairs. The commissure is wide. The hollow secretory tubules are solitary, thin, and slightly widened at the bottom to 0.3 mm. The endosperm is flat on the commissural side. The weight of 1000 seeds is 8–10 g, and dry seed productivity can reach values of 120–150 g/plant [8].

The roots and seeds of *H. dissectum*, which are known as *balchirgana* (Buryat, Mongolian, and Manchurian names), *istii ot* or *puchka* (Yakutian name), *spru ma* (Tibetan name), and *gao ben*’ (Chinese name), have a bitter and pungent taste and are used in Asian medicines as drugs [9]. Buryat lamas cure furunculus, ulcers, bleedings, anemia, and wounds by hogweed decoctions [10], and the oil of hogweed seeds is used as a remedy against noise in ears, vertigo, and headache [11]. Yakutian healers use hogweed as an appetizer, spasmolytic, and antiseptic drug, as well as a component of the nervous system and skin disease treatments [12]. In Chinese Traditional Medicine, the plant is used for dispelling wind, eliminating dampness, and curing rheumatoid diseases, waist or knee pain, and headaches [13].

The known phytocomponents [13,14,15,16,17,18,19,20,21,22,23] found in the roots and herb of *H. dissectum* include aliphatic compounds [14,15], terpenes [14,16,21], and phenolics [13,14,17,18,19,20,21,22,23] (Table 1).

The basic phenolic group of *H. dissectum* is coumarins, including simple coumarins [18,20,21,22,23], linear and angular furanocoumarins, dihydrofuranocoumarins, and dihydropyranocoumarins [13,15,16,18,19,20,21,22]. The analysis of essential oils of herbs [24], lamina, and petiole [25] has shown the presence of monoterpenes, sesquiterpenes, aliphatic alcohols, and esters. To date, there is no information on metabolites in *H. dissectum* seeds.

The most diverse metabolites found in *H. dissectum* are coumarins, which are a group of phytocompounds with anti-inflammatory [26], anti-HIV [27], antimicrobial [28], anticancer [29], and antiviral properties [30]. A distinctive feature of coumarins is their lipophilicity [31], which makes it difficult to dissolve or disperse them in safe and water-based solvents that could be used in experiments on living organisms. To solve the problem of insolubility of coumarins in water, the use of nanosuspensions has been proposed and recently studied as the most promising strategy to enhance the oral bioavailability of these types of drugs [32,33]. Nanosuspensions have been used to enhance the bioavailability of curcumin [34], cannabidiol [35], naringenin [36], daidzein [37], and other bioactive molecules as well as plant-derived fatty materials such as olive leaf extract [38], *Rauwolfia serpentina* extract [39], and fennel seed extract [40].

The aim of this study is the investigation of the seed oil of *H. dissectum* (HSO) by the physico–chemical methods, high-performance liquid chromatography with photodiode array detection, and electrospray ionization triple quadrupole mass spectrometric detection (HPLC–PDA–ESI–tQ–MS/MS) profiling and quantification as well as investigation of neuroprotective potential of the nanosuspension of HSO obtained using CO_2_-assisted effervescence method in brain ischemia model of rats.

## 2. Materials and Methods

### 2.1. Plant Material and Chemicals

**Figure 1:**
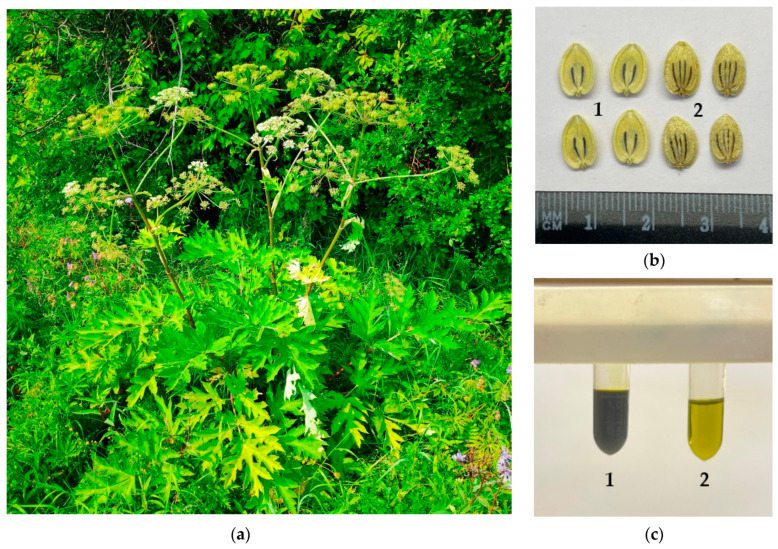
(**a**) Dissected hogweed (*Heracleum dissectum* Ledeb.) in the natural habitat (Kharashibir vicinity, Mukhorshibir District, Buryatia Republic, Russia). (**b**) Hogweed seeds (b_1_: inner side; b_2_: outer side). (**c**) Hogweed seed oil raw (c_1_) and diluted with olive oil (1:10; c_2_).

Ripe seeds of *Heracleum dissectum* were collected in the Mukhorshibir vicinity (Mukhorshibirskii District, Buryatia Republic, Russia; Figure 1a,b; 51°03′58.2″ N 107°55′00.2″ E, 790 m a.s.l.; sample 1, collection date 29 August 2019, voucher No BUR/API-0819/59-365; sample 2, collection date 28 August 2020, voucher No BUR/API-0820/63-211; sample 3, collection date 30 August 2021, voucher No BUR/API-0821/83-407; sample 4, collection date 29 August 2022, voucher No BUR/API-0820/76-416). Samples were authenticated by Prof. N.I. Kashchenko (IGEB SB RAS, Ulan-Ude, Russia). The fresh seeds were conditioned in plastic boxes and transported to the laboratory within 3–4 h, where they were dried in the ventilated heat oven at 35 °C within 5–7 days and stored at 4–6 °C before manipulations. The reference standards were purchased from AbMole BioScience (Houston, TX, USA); AOBIOUS Inc. (Gloucester, MA, USA); BenchChem (Austin, TX, USA); BioCrick (Chengdu, Sichuan, China); MCE Med Chem Express (Monmouth, NJ, USA); Sigma-Aldrich (St. Louis, MO, USA); and Selleck Chemicals (Houston, TX, USA) (Table S1).

### 2.2. Seed Oil Preparation

Dry and milled seeds (1 kg; sample 1) were exhausted extracted in Soxhlet extractor (internal volume 2 L; Borosil^®^ Extraction Apparatus, Foxx Life Sciences, Salem, NH, USA) with petroleum ether (boiling point 30–40 °C; Sigma-Aldrich; cat. No 77399). The organic extract was concentrated in a vacuum at 30 °C to give colored viscous oil with a specific odor (yield 105.2 g) stored under nitrogen at 0 °C before manipulations.

### 2.3. Seed Oil Physico–Chemical Analysis

The following physical parameters of *H. dissectum* seed oil were determined: viscosity—viscosimetric method [41] at 20 °C using Cannon-Fenske Routine Viscometer (Cannon Instrument Company, State College, PA, USA); specific gravity—picnometric method [42] at 20 °C using PZh2-5-KSh 7/16 picnometer (MiniMed Ltd., Suponevo, Russia); refractive index—refractometric method AOAC 921.08 (Refractive Index of Edible Oils and Fats) [43] at 20 °C using Atago 3454 PR-butyro digital butyro refractometer (Atago, Tokyo, Japan); pH—potentiometric method [44] at 20 °C using Thermo Scientific Orion Versa Star Multi-parameter Benchtop Meter (Thermo Fisher Scientific Inc, Waltham, MA, USA); melting point—differential scanning calorimetric method [45] using STA 449C C/4/G Jupiter thermo gravimetric analyzer (Netzsch, Selb, Germany). The chemical parameters were determined using AOAC assays [43] as peroxide value (AOAC 965.33), acid value (AOAC 940.28), saponification value (AOAC 41.1.18), iodine value (AOAC 41.1.15), and unsaponifiable matter (AOAC 975.13). Spectrophotometric assays were used to determine content of chlorophylls and carotenoids [46], and coumarins [47]. Essential oil content in *H. dissectum* seed oil was determined after the hydrodistillation procedure in the Clevenger apparatus with a 10 g-sample of the oil [48]. The composition of fatty acids and essential oil was analyzed by gas chromatography-mass spectrometric procedure described previously [49] using Agilent 6890 N gas chromatograph and an Agilent Technologies 5973 N mass selective/quadrupole detector (Agilent Technologies Inc., Santa Clara, CA, USA).

### 2.4. Ultraviolet Spectroscopy of Seed Oil

The seed oil of *H. dissectum* (250 mg) was transferred to the volumetric flask (25 mL), diluted in acetonitrile, and the total volume was filled to 25 mL (solution A; 10 mg/mL). An aliquot of solution A (100 μL) was diluted in the volumetric flask (25 mL) by acetonitrile (solution B; 40 μg/mL). Ultraviolet spectra of solutions A and B were studied using an SF-2000 spectrophotometer (Specter, St. Petersburg, Russia) in 1 cm-quartz cells and pure acetonitrile as a blank [50]. Imperatorin solution in acetonitrile was used as a reference standard with the final concentration of 10 μg/mL.

### 2.5. Fourier-Transform Infrared Spectroscopy (FTIR) of Seed Oil

FTIR spectra of *H. dissectum* seed oil were studied using FT-801 Fourier-transform infrared spectrometer (Simex, Novosibirsk, Russia; frequency 600–4000 cm^−1^, 200 scans, 2-cm^−1^ resolution) coupled with attenuated total reflection device (ATR) [51].

### 2.6. High-Performance Liquid Chromatography with Photodiode Array Detection and Electrospray Ionization Triple Quadrupole Mass Spectrometric Detection (HPLC-PDA-ESI-TQ-MS) Metabolite Profiling of Seed Oil

To profile coumarins in *H. dissectum* seed oil, the HPLC-PDA-ESI-TQ-MS method was performed using the liquid chromatograph LC-20 Prominence coupled with photodiode array detector SPD-M30A, triple-quadrupole mass spectrometer LCMS 8050, and GLC Mastro column (2.1 mm × 150 mm × 3 μm; all Shimadzu, Kyoto, Japan). Separation was provided in the gradient mode by means of eluent A (1% formic acid in water) and B (1% formic acid in acetonitrile) and the gradient program (%B): 0–2 min 5–15%, 2–8 min 15–27%, 8–20 min 27–80%, and 20–29 min 80–100%, 29–35 min 100–5%. The parameters of injection volume, flow rate, and column temperature were 1 μL, 200 μL/min, and 27 °C, respectively. A spectral range of 200–600 nm was used to record ultraviolet spectra. Temperature levels of electrospray ionization triple quadrupole mass spectrometric detection ESI interface, desolvation line, and heat block were 300 °C, 250 °C, and 400 °C, respectively, and the values of nebulizing gas (N_2_) flow, heating gas (air) flow, and collision-induced dissociation gas (Ar) glow were 3 L/min, 10 L/min, and 0.3 mL/min, respectively. Electrospray ionization was done with scanning range *m*/*z* 80–1900, source voltage 3 kV, and collision energy +25 eV (positive ionization). To manage the LC-MS system, the preinstalled software LabSolutions LCMS ver. 5.6 [52] was used. Metabolite identification was realized after integrated analysis of chromatographic parameters (retention time) and spectral data (ultraviolet pattern, mass spectra) after comparison with the inner LC-MS library, reference standards, and the literature data. To prepare the sample solution, *H. dissectum* seed oil (25 mg) was dissolved in acetonitrile in a measuring flask (5 mL), followed by filtering through 0.22 μm syringe filters.

### 2.7. HPLC-ESI-TQ-MS Quantification of Coumarins in Seed Oil

Six coumarins (heraclenin, oxypeucedanin, imperatorin, phellopterin, isoimperatorin, and ostruthin) were quantified using HPLC-ESI-TQ-MS conditions described in Section 2.6. Separately weighed reference standards (10 mg) were dissolved in acetonitrile in volumetric flasks (10 mL), and the stock solutions (1000 µg/mL) were applied for preparation of the calibration solutions (1–100 µg/mL) and creation of correlations ‘concentration–mass spectral peak area’. The values of correlation coefficient (r*^2^*), standard deviation (S_YX_), the limit of detection (LOD), limit of quantification (LOQ), and linear range were calculated in Advanced Grapher 2.2 (Alentum Software Inc., Ramat-Gan, Israel) using calibration curve data [53] and the results of three sufficient HPLC runs (Table 2). Iintra-day, inter-day precisions, and recovery of spiked samples were studied using the known assay [54]. The results were expressed as mean values ± standard deviation (S.D.).

### 2.8. Heracleum dissectum Seed Oil Storage Experiment

Three aliquots of *H. dissectum* seed oil (sample 1; 10 mL) were placed in the individual polystyrene tubes and thermostated at (1) 20 °C, 1 °C, and −20 °C for three years using a ventilated MK 53 thermostat (BINDER GmbH, Tuttlingen, Germany) [55]. One stored sample was taken out for analysis every year and studied by the preparation/analysis procedure described in Section 2.6.

### 2.9. Nanosuspension of H. dissectum Seed Oil Preparation

The early recommendations were used to prepare *H. dissectum* seed oil nanosuspension [40] based on the CO_2_-assisted effervescence technique [56]. The mixture of *H. dissectum* seed oil (20 mg), citric acid (30 mg), and tocopheryl polyethylene glycol succinate (20 mg) was dissolved in 50 mL of ethyl acetate, and the organic solvent was evaporated in a vacuum. The residue was mixed with 50 mL of NaHCO_3_ solution (0.08%) and vigorously stirred for 20 min.

### 2.10. Characterization of H. dissectum Seed Oil Nanosuspension

Particle size, polydispersity index distribution, and zeta potential were studied using Dynamic Light Scattering Zetasizer Nano ZS (Malvern Instruments, Malvern, UK) at 20 °C (laser wavelength 633 nm) [57]. All measurements were performed three times.

### 2.11. Neuroprotective Activity

An animal model of brain ischemia was used to study the neuroprotective activity of *H. dissectum* seed oil nanosuspension performed as described early [58]. In brief, permanent focal cerebral ischemia of rats was reproduced by right-sided thermocoagulation of the middle cerebral artery in six animal groups (*n* = 15), including (1) sham-operated animals; (2) negative control group with animals after focal cerebral ischemia without pharmacological support; (3) EGB761 group with animals after focal cerebral ischemia treated with a reference drug EGB761 (*Ginkgo biloba* extract, Hunan Warrant Pharmaceuticals, Changsha, China; 35 mg/kg [59]; (4, 5, 6) HSO 0.1, 0.5, 1.0 mL/kg groups of animals after focal cerebral ischemia treated with *H. dissectum* seed oil nanosuspension in doses 0.1, 0.5, 1.0 mL/kg. After the 4-day-treatment, an average systolic velocity of cerebral blood flow was determined using an ultrasound Doppler device, a sensor USOP-010-01 with a working frequency of 25 MHz, and an MM-D-K-Minimax Doppler v.1.7. (Saint Petersburg, Russia) [60] followed by the animal’s decapitation, brain extraction, and measuring the area of necrosis zone. All measurements were performed once for each animal and in total 15 times for one experimental group.

### 2.12. Statistical Analysis

All quantitative analyses were performed five times, and the data were expressed as the mean value ± standard deviation (S.D.). Statistical analyses were performed by one-way analysis of variance, and the significance of the mean difference was determined by Duncan’s multiple-range test. Differences at *p* < 0.05 were considered statistically significant. The linear regression analysis and generation of calibration graphs were conducted using Advanced Grapher 2.2 (Alentum Software, Inc., Ramat-Gan, Israel).

## 3. Results and Discussion

### 3.1. Physico–Chemical and Spectral Characteristics of Heracleum dissectum Seed Oil

The oil from *Heracleum dissectum* seeds (HSO) was obtained with a yield of 10.52% (Table 3); it is a mobile liquid, yellow to green–yellow in color, with specific hogweed fragrance (Figure 1c).

The values of specific gravity and refractive index are 0.929 g/mL and 1.472, respectively, and are similar to those of carrot seed oil (0.981 g/mL, 1.473) [61], *Momordica charantia* seed oil (0.998 g/mL, 1.500) [62], and pumpkin seed oil (0.96 g/mL, 1.47) [63]. The level of pH is 6.20, which indicates the neutrality of oil, which is similar to those of seed oils of sesame (pH 6.12), melon (pH 6.42), or morinda (pH 6.78) [64]. The peroxide value of HSO is 6.28 mEq. peroxide/kg, which is considerably below that of carrot seed oil (16.0 mEq./kg) [61], higher than that of jatropha seed oil (0.8–1.9 mEq./kg) [65], and similar to that of sunflower seed oil (6.8–7.2 mEq./kg) [66]. The acid value of HSO is 0.52 mg KOH/g, and the saponification value is 173.82 mg KOH/g, which is similar to those of pumpkin seed oil (0.57–0.64 mg KOH/g; 189–190 mg KOH/g) [63]. However, the iodine value of HSO is 105.37 g I_2_/100 g, which indicates the high level of unsaturation that is typical for sunflower oil (118–141 g I_2_/100 g), sesame oil (103–120 g I_2_/100 g), and rice bran oil (90–115 g I_2_/100 g) [67]. Unsaponifiable matter level (0.92%) was similar to those of apiaceous seed oils from the carrot (0.9%), dill (1.2%), coriander (2.2%), and caraway (2.5%) [68]. The low melting point (−25.3 °C) allows HSO to remain a liquid even at low temperatures.

**Figure 2:**
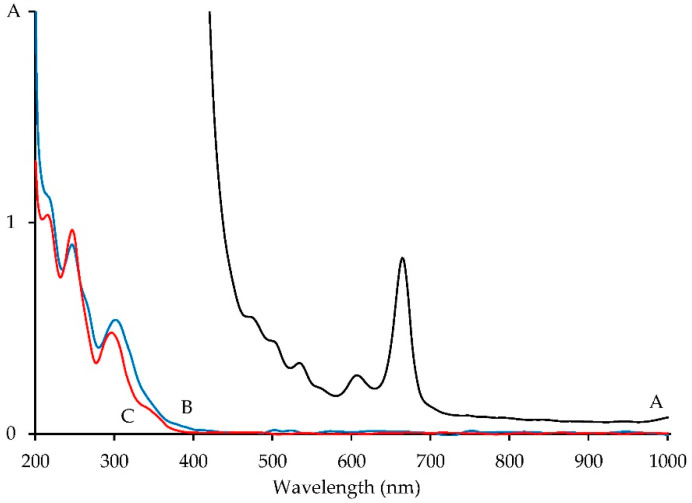
UV-Vis spectrum of *H. dissectum* seed oil solutions in acetonitrile (A: 10 mg/mL; B: 40 μg/mL) and imperatorin solution in acetonitrile (C: 10 μg/mL).

The seeds of *H. dissectum* are weakly pigmented, which results in the dark color of the oil. The absorption spectrum of HSO demonstrated the presence of long-wave bands at 600–700 and 450–550 nm, which are caused by chlorophylls and carotenoids from seed coats [69] (Figure 2). The concentration of chlorophylls and carotenoids in HSO is 364.08 and 233.94 mg/L, respectively. The known plant oil composition data indicate a lower pigment content in olive oil (4.9–24.4 mg/L of chlorophylls and 3.1–13.4 mg/L of carotenoids [70]) or in grape seed oil (1.0–3.8 mg/L of chlorophylls and 2.6–4.8 mg/L of carotenoids [71]).

The main fatty acid components of HSO are petroselinic acid (*cis*-6-octadecenoic acid; 48.3%), linoleic acid (*cis*,*cis*-9,12-octadecadienoic acid; 28.3%), oleic acid (*cis*-9-octadecenoic acid; 10.2%), and palmitic acid (hexadecanoic acid; 5.2%); their total content reaches 92%. The domination of these compounds in seed oils is a distinctive feature of the *Heracleum* genus. European species (such as *H. montanum*, *H. orphanidis*, *H. pyrenaicum subsp. orsinii*, *H. pyrenaicum subsp. pollinianum*, *H. sibiricum*, *H. sphondylium*, *H. ternatum*, and *H. verticillatum*) demonstrate the prevalence of petroselinic acid in the range of 42.8–56.5% [72]. The content of linoleic and oleic acids in the specified plants is 20.3–33.3% and 12.3–13.7%, respectively.

The intense fragrance of HSO indicates the presence of volatile components whose content after hydrodistillation is 32.31% of HSO weight. The results of GC–MS analysis of essential oil revealed the presence of 21 compounds, including octyl acetate (67.8%), octyl 2-methyl isobutyrate (9.6%), and hexyl 2-methylbutyrate (8.5%) as basic components (Table 4). Octyl acetate has a fruity, slightly fatty, waxy, floral odor [73]; these characteristics best describe the HSO odor. The main components of the essential oils distilled from the seeds of other *Heracleum* species are octyl acetate (39.5%), hexyl 2-methylbutyrate (14.4%), octanol (8.6%), and hexyl 2-methylpropanoate (6.0%) in *H. sosnowskyi* [74]; hexyl butyrate (20.9–44.7%) and octyl acetate (11.2–27%) in *H. persicum* [75,76]; octyl acetate (69.4–76.5%) and hexyl butyrate (3.2–6.2%) in *H. anisactis* [77]; 1-octanol (50.3%), octyl butyrate (24.6%), and octyl acetate (7.3%) in *H. sphondylium* subsp. *ternatum* [78]. It is possible that octanol and hexanol and its esters are typical for the genus *Heracleum*.

The dilution of HSO led to the formation of a specific spectral pattern in the UV region, which was similar to those of 8-*O*-substituted furanocoumarins [79] and indicated the presence of these phytocomponents (Figure 2). The spectrophotometric assay allowed us to determine that the total content of coumarins in HSO was approximately 24.52%, which is characterized as a high level.

**Figure 3:**
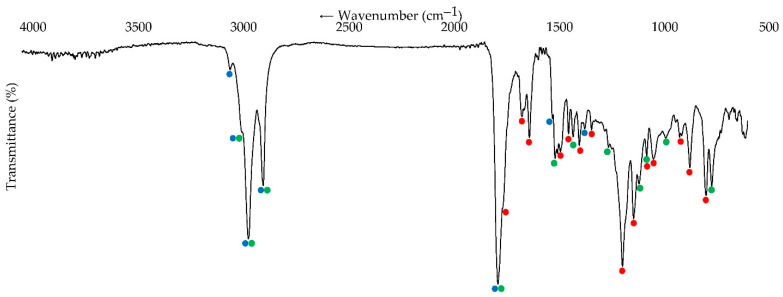
FTIR spectrum of *H. dissectum* seed oil. Colored circles indicate bands caused by the possible presence of fatty acids (blue), essential oil components (green), and coumarins (red).

For the further study of HSO, Fourier-transform infrared spectroscopy was applied, which is a commonly used method for the analysis and authentication of edible oils [80]. The spectral pattern of HSO is complex and characterized by various bands, which were assigned to three groups of phytocompounds after comparison with the reference compounds such as petroselinic acid (fatty acid example), octyl acetate (essential oil component), and imperatorin (furanocoumarin example) (Figure 3 and Figure S1). The most intense bands were attributed to the fatty acids, specifically C-H stretching of H-C=C at 2921 cm^−1^, C-H symmetric stretching at 2851 cm^−1^, C=O stretching at 1736 cm^−1^, and C-H bending at 1463 cm^−1^ [81]. The alkyl fragment of octyl acetate gave the bands from the same “fatty” regions together with specific bands caused by acetate and octyl fragments at 1378, 1211, 1066, 1029, 938, and 721 cm^−1^ [82]. Bands of furanocoumarins clearly appeared at 700–1800 cm^−1^, more specifically at 1713 (lactonic C=O), 1621 (furanic C=C), 1586, 1440 (aromatic C=C, 8-*O*-substituted furanocoumarins), 1324 (aryl-O of methoxylated coumarins), 1144, 1093 (furan ring), 997, 874, 825, and 748 cm^−1^ (deformation vibrations of C-H) [83]. The FTIR spectrum of HSO allows for elucidating the general composition of seed oil because it contains the bands of all dominant phytocomponents.

### 3.2. Coumarin Profile of Heracleum dissectum Seed Oil

**Figure 4:**
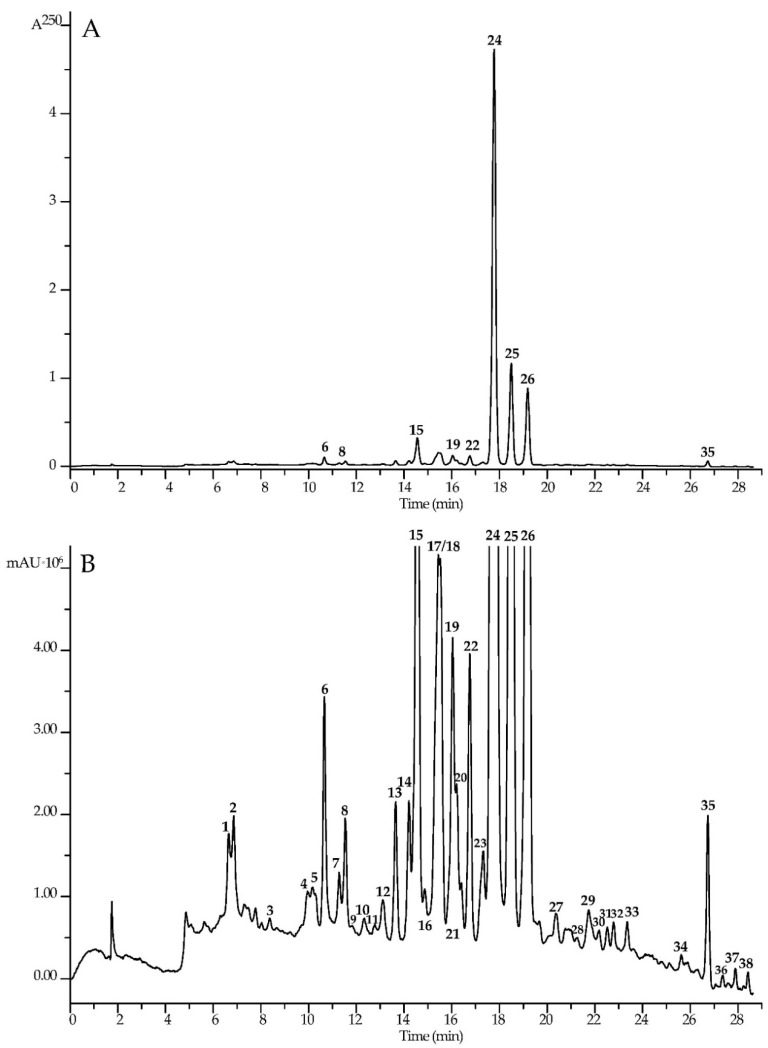
High-performance liquid chromatography data of *H. dissectum* seed oil solution (1 mg/mL in MeCN) with photodiode array detection ((**A**); 250 nm) and electrospray ionization triple quadrupole mass spectrometric detection ((**B**); positive ionization detection, base peak chromatogram). Compounds are numbered as listed in Table 5.

**Figure 5:**
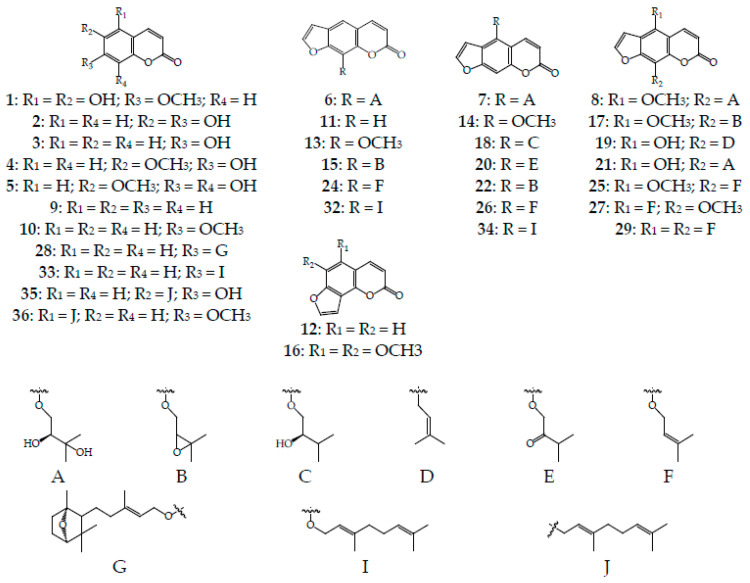
Structures of compounds found in *H. dissectum* seed oil.

The high coumarin content in HSO allowed us to realize profiling by HPLC–PDA–ESI–tQ–MS/MS. This led to the discovery of 38 compounds, which were identified on the basis of retention times as well as UV and mass spectrometric data after comparison with reference substances and literature data [84,85,86,87] (Figure 4, Table 4). The structures of thirty-two coumarins were accurately identified, and the structures were predicted for six compounds (Figure 5).

**Table 5:** Chromatographic (t) and ultraviolet (UV) and mass-spectrometric (ESI-MS) data of compounds **1**–**38** found in *H. dissectum* seed oil.

No.	t, min	UV, λ_max, nm_	ESI-MS, *m*/*z* (I, %)			Compound [Ref.]	IL *
			[M + H]^+^	[M + Na]^+^	[M + K]^+^		
**1**	6.22	211, 229, 344	209 (11)	231 (100)	247 (54)	Isofraxetin [84]	1
**2**	6.93	230, 259, 302, 345	179 (9)	201 (100)	217 (63)	Esculetin [85]	1
**3**	8.19	216, 324	163 (22)	185 (100)	201 (58)	Umbelliferone [86]	1
**4**	9.92	228, 230, 300, 342	193 (4)	215 (100)	231 (69)	Scopoletin [84]	1
**5**	10.05	230, 261, 345	209 (10)	231 (100)	247 (82)	Fraxetin [85]	1
**6**	10.72	217, 248, 265, 300	305 (8)	327 (100)	343 (42)	Heraclenol (prangenin hydrate, komaline) [84]	1
**7**	11.29	222, 250, 265, 308	305 (11)	327 (100)	343 (38)	Oxypeucedanin hydrate (prangol) [84]	1
**8**	11.62	222, 249, 267, 311	335 (26)	357 (100)	373 (73)	Byakangelicin [84]	1
**9**	11.88	272, 310	147 (4)	169 (100)	185 (56)	Coumarin [84]	1
**10**	12.28	214, 300, 318	177 (11)	199 (100)	215 (36)	Herniarin [84]	1
**11**	12.82	244, 292, 330	187 (12)	209 (100)	225 (49)	Psoralen [87]	1
**12**	13.11	200, 216, 258, 302	187 (3)	209 (100)	225 (72)	Angelicin [87]	1
**13**	13.72	217, 247, 265, 301	217 (8)	239 (100)	255 (53)	Xanthotoxin [87]	1
**14**	14.15	222, 248, 268, 312	217 (5)	239 (100)	255 (42)	Bergapten [87]	1
**15**	14.63	215, 250, 265, 305	287 (18)	309 (100)	325 (56)	Heraclenin (prangenine) [84]	1
**16**	14.92	217, 247, 265, 300	247 (26)	269 (100)	285 (73)	Pimpinellin [87]	1
**17**	15.34	221, 247, 267, 310	317 (5)	339 (100)	355 (42)	Byakangelicol [84]	1
**18**	15.37	220, 251, 266, 304	289 (2)	311 (100)	327 (39)	Pranferol [85]	1
**19**	16.03	220, 248, 268, 311	271 (15)	293 (100)	309 (73)	Alloimperatorin (prangenidin) [84]	1
**20**	16.14	220, 251, 265, 304	287 (12)	309 (100)	325 (31)	Isooxypeucedanin [84]	1
**21**	16.42	220, 248, 267, 312	319 (9)	341 (100)	357 (39)	Heracol [84]	2
**22**	16.82	217, 248, 266, 301	287 (5)	309 (100)	325 (63)	Oxypeucedanin (prangolarlin) [84]	1
**23**	17.22	217, 248, 267, 300	287 (3)	309 (100)	325 (40)	Oxypeucedanin isomer [84]	2
**24**	17.73	217, 248, 265, 300	271 (11)	293 (100)	309 (70)	Imperatorin (ammidin, marmelosin) [84]	1
**25**	18.51	221, 248, 268, 311	301 (26)	323 (100)	339 (72)	Phellopterin [85]	1
**26**	19.12	220, 250, 265, 305	271 (7)	293 (100)	309 (65)	Isoimperatorin [87]	1
**27**	20.27	221, 249, 267, 312	301 (14)	323 (100)	339 (51)	Cnidilin (isophellopterin) [87]	1
**28**	21.18	224, 326	383 (6)	405 (100)	421 (63)	Farnesiferol C [85]	1
**29**	21.80	220, 248, 267, 312	355 (8)	377 (100)	393 (52)	Cnidicin [84]	1
**30**	22.09	216, 247, 265, 301	299 (10)	321 (100)	337 (45)	Auraptene isomer [84]	2
**31**	22.53	220, 247, 267, 311	339 (7)	361 (100)	377 (83)	Bergamottin isomer [84]	2
**32**	22.82	216, 247, 265, 301	339 (12)	361 (100)	377 (46)	8-Geranyloxypsoralen [84]	1
**33**	23.22	220, 325	299 (8)	321 (100)	337 (40)	Auraptene [84]	1
**34**	25.71	220, 247, 267, 311	339 (5)	361 (100)	377 (80)	Bergamottin [84]	1
**35**	26.64	220, 324	299 (12)	321 (100)	337 (63)	Ostruthin [85]	1
**36**	27.28	219, 267, 325	313 (10)	335 (100)	351 (61)	5-Geranyloxy-7-methoxycoumarin [84]	1
**37**	27.91	220, 324	299 (14)	321 (100)	337 (60)	Ostruthin isomer [85]	2
**38**	28.47	219, 267, 325	313 (11)	335 (100)	351 (53)	5-Geranyloxy-7-methoxycoumarin isomer [84]	2

* Identification level: (1) identified compounds after comparison of UV, mass-spectral data, and retention time with reference standards; (2) putatively annotated compounds after comparison of UV and mass-spectral data with literature data.

Eleven identified compounds are simple coumarins that are based on the benzopyran-2-one substituted at C-5, C-6, C-7, and C-8 positions including unsubstituted coumarin (**9**) and its derivatives with simple substituents (hydroxyl and methoxyl) such as umbelliferone (**3**) and scopoletin (**4**), which have been previously detected in *H. dissectum* roots [18,20] and herb [21], and new hogweed coumarins herniarin (**10**), esculetin (**2**), isofraxetin (**1**), and fraxetin (**5**). Compounds **28** and **33** are derivatives of umbelliferone (7-hydroxycoumarin, **3**); farnesiferol C (**28**) is a sesquiterpene coumarin found in *Ferula* genus [88], and auraptene (**33**) or 7-geranyloxycoumarin is typical for *Ferula* and *Citrus* species [89].

Esculetin derivative ostruthin (**35**) or 6-geranyloxy-7-hydroxycoumarin has been previously identified in *Peucedanum* genus [90], and 5-geranyloxy-7-methoxycoumarin is a component of bergamot essential oil [91]. Compounds **28**, **33**, **35**, and **36** are the new *Heracleum* genus metabolites. The mass spectral patterns of coumarins **30**, **37**, and **38** are similar to those of auraptene, ostruthin, and 5-geranyloxy-7-methoxycoumarin, respectively, and the compounds are isomers.

The remaining compounds are furanocoumarins with linear and angular molecular geometry. Linear furanocoumarins in *H. dissectum* seed oil are derivatives of psoralen (**11**) and are divided into three types of substitution, including 5-*O*-, 8-*O*-, and 5,8-di-*O*-substitution. Coumarins with a functional group only at the C-5 position are bergaptol esters (5-hydroxypsoralen) and identified as oxypeucedanin hydrate (prangol, **7**), bergapten (**14**), pranferol (**18**), isooxypeucedanin (**20**), oxypeucedanin (prangolarlin, **22**), isoimperatorin (**26**), and bergamottin (**34**). Bergapten has been previously found only in the roots of *H. dissectum* [13,20], isoimperatorin has been identified in the fruits of *H. leskowii* [92], while **7**, **18**, **20**, **22**, and **34** have been detected for the first time for these species and genus. Among the 8-*O*-substituted furanocoumarins are: a) esters of xanthotoxol (8-hydroxypsoralen), which has been previously isolated from the roots of *H. dissectum*; the identified esters include heraclenol (prangenin hydrate, komaline, **6**) [15,16,23], xanthotoxin (**13**) [13], and imperatorin (**24**) [13]; b) heraclenin (prangenin, **15**), which has been detected in *H. candicans* [93], *H. canescens* [94], and *H. sibiricum* [95]; and c) 8-geranyloxypsoralen (**32**), which has been found in *H. candicans* [93].

Seven 5,8-di-substituted furanocoumarins are known herb coumarins of *H. dissectum* including byakangelicin (**8**) [21] and phellopterin (**25**) [21] in addition to alloimperatorin (prangenidin, **19**), which has been detected in *H. canescens* [94], and heracol, which has been isolated from the roots of *H. leskowii* [96] and *H. pastinacifolium* [97]. Byakangelicol (**17**), cnidilin (isophellopterin, **27**), and cnidicin (**29**) have not been previously detected in the *Heracleum* genus. Two known *Heracleum* furanocoumarins with angular skeletons of angelicin (**12**) and pimpinellin (**16**) have been detected in *H. dissectum* roots [13,20,22] and herb [21] and in other hogweeds such as *H. leskowii* [92], *H. mantegazzianum* [98], and *H. maximum* [99].

The obtained data allow us to conclude that *H. dissectum* seed oil is a source of coumarins that are typical for the *Heracleum* genus [6] and Apiaceae family as a whole [100], and some simple coumarins and furanocoumarins have been newly detected.

### 3.3. Quantification of Six Coumarins in Heracleum dissectum Seed Oil before and after Storage

To further characterize coumarins in HSO, quantification of six dominant compounds was performed by HPLC-ESI-TQ-MS, which allowed to determine the concentrations of heraclenin, oxypeucedanin, imperatorin, phellopterin, isoimperatorin, and ostruthin (Table 6).

The variation of coumarin content in four samples of HSO was 5.14–10.48 mg/mL for heraclenin, 0.93–5.76 mg/mL for oxypeucedanin, 108.83–153.05 mg/mL for imperatorin, 30.83–42.10 mg/mL for phellopterin, 11.67–29.52 mg/mL for isoimperatorin, and 4.59–5.09 mg/mL for ostruthin. The total coumarin content in samples was 181.14–238.42 mg/mL. Imperatorin is a dominant coumarin in *H. dissectum* seed oil and, as has been shown earlier, in *H. leskowii* seed lipophilic fractions [101] and *H. verticillatum* seed extract [102].

Owing to the lipophilic nature of *H. dissectum* seed oil, the storage of HSO may lead to a loss of quality parameters, including coumarin content. Therefore, it is helpful to study the stability of marker compounds under various storage conditions, i.e., room, cold, and freezing temperatures (Table 6). The 3-year room temperature storage of HSO resulted in the greatest loss of total coumarin content, i.e., 8.9% after 1-year storage, 16.2% after 2-year storage, and 27.9% after 3-year storage. A decrease in storage temperature helped to preserve coumarins in HSO; specifically, after 3-year storage at 1 °C and at −20 °C, the total coumarin recovery was 92.9% and 97.7% of the initial content, respectively. This is a clear indication of the value of storage temperature on the quality of seed oil.

### 3.4. Nanosuspension of Heracleum dissectum Seed Oil and Its Neuroprotective Activity

**Figure 6:**
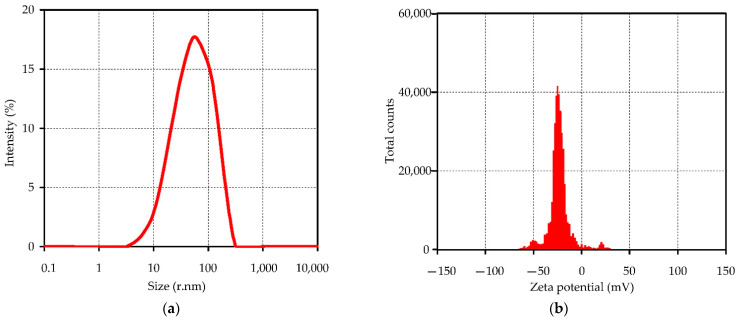
Particle size and polydispersity index distribution (**a**) and zeta potential (**b**) of *H. dissectum* seed oil nanosuspension.

Among the many existing methods of nanosuspension preparation, the CO_2_-assisted effervescence method was successfully applied to *H. dissectum* seed oil [103]. Prepared HSO nanosuspension has small particles (82.36 nm, polydispersity index 0.208), and zeta potential showed surface charge values of −25.3 mV (Figure 6), which indicates that this formulation is characterized by nanometer-scale particles and homogenous dispersion [104].

Permanent focal cerebral ischemia caused by the right-sided thermocoagulation of the middle cerebral artery in rats reduces cerebral blood flow (1.25 sm/s vs. 4.10 sm/s in the sham-operated group; *p* < 0.05) and increases necrosis zone area to 41.52% (Table 7). The application of a standardized extract of *Ginkgo biloba* (EGB761) demonstrated a positive effect characterized by increased cerebral blood flow (2.63 sm/s; *p* < 0.05) and reduction of necrosis zone area down to 21.60% (*p* < 0.05), which is typical for the plant extracts with neuroprotective effects such as *Ginkgo biloba* [105], *Rhaponticum uniflorum* [106], *Serratula centauroides* [107], and *Nepeta multifida* [108]. The nanosuspension of HSO in doses of 0.1–1 mL/kg demonstrated a positive dose-dependent effect, which increased with dose. The most pronounced neuroprotective activity was found for the dose of 1 mL/kg, which increased cerebral blood flow to 3.11 sm/s (*p* < 0.05) and decreased necrosis zone area to 18.56% (*p* < 0.05); this result indicates the greater therapeutic effect of HSO compared to that of the EGB761 reference drug.

The know literature data indicate that the selected components of *H. dissectum* seed oil have a great influence on the ischemic brain tissues. The basic coumarin of the plant, imperatorin, protects the brain against extreme oxidative stress induced by cerebral ischemia/reperfusion in rats through activation of the Nrf2 signaling pathway [109] and/or anti-apoptosis function [110]. Imperatorin reduces neuronal apoptosis and boosts synaptic plasticity in a vascular dementia model of rats developed by the modified ligation of perpetual two-vessel occlusion [111]. Imperatorin performed an anti-inflammatory role through the downregulation of MAPK and NF-κB signaling pathways in ischemic stroke-induced to microglia-mediated neuroinflammation and was determined to be a potential anti-stroke agent [112]. Imperatorin demonstrates a significant vasorelaxant activity (which is higher than that of acetylcholine), radical scavenging [113], and antidepressant potential [114]. Geranylated coumarin ostruthin, owing to its TREK-1 channel activator activity, showed antidepressant and anxiolytic effects in mice evaluated by the open-field, elevated plus maze, and light/dark box tests [115]. Unsaturated fatty acids can protect the brain against ischemic injury by activating Nrf2 and upregulating heme oxygenase 1 [116]. Perhaps other phytocomponents of *H. dissectum* seed oil may have positive effects on the ischemic brain; however, this question will be addressed in future studies.

## 4. Conclusions

This study demonstrated for the first time that *H. dissectum* seeds are a source of valuable oil. Physico–chemical parameters and phytocompounds present in seed oil (HSO) were characterized by various methods, including high-performance liquid chromatography with photodiode array detection and electrospray ionization triple quadrupole mass spectrometric detection. Fatty acids, volatile components, coumarins, and photosynthetic pigments were found in HSO and quantified. Coumarins were separated by the LC–MS technique, and HSO was determined to be a source of furanocoumarins among which heraclenin, oxypeucedanin, imperatorin, phellopterin, isoimperatorin, and ostruthin were predominant with the total content of 18.1–23.8%. Stability study showed that cold and freezing storage resulted in the best preservation of coumarins in HSO. Our findings suggest that it is possible to obtain HSO nanosuspensions with neuroprotective activity, as determined using the model of cerebral ischemia in rats. Thus, *H. dissectum* is a bioactive plant. These results will help create new nanotherapeutic remedies.

## Figures and Tables

**Table 1:** Synopsis of known hogweed (*Heracleum dissectum* Ledeb.) metabolites.

Compound ^a^	Organ (Origin ^b^) [Ref.]
Alkyl glycosides	
*n*-Butyl-*O*-Frc*p*	Roots (CHI) [14]
Polyynes	
Falcarindiol	Roots (CHI) [15]
4,6-Decadiyne 1-*O*-(2′-*O*-(6″-*O*-Glc*p*)-Glc*p*)-Glc*p*	Roots (CHI) [15]
(8*Z*)-Decaene-4,6-diyn 1-*O*-(2′-*O*-(6″-*O*-Glc*p*)-Glc*p*)-Glc*p*	Roots (CHI) [15]
(8*E*)-Decaene-4,6-diyn 1-*O*-(2′-*O*-(6″-*O*-Glc*p*)-Glc*p*)-Glc*p*	Roots (CHI) [15]
Semiterpene glycosides	
Butane-2,3-diol 2-*O*-Glc*p*	Roots (CHI) [14]
2-Methyl-1-butanol 1-*O*-Glc*p*	Roots (CHI) [14]
3-Methyl-1-butanol 1-*O*-Glc*p*	Roots (CHI) [14]
3-Methylbutan-1,3-diol 1-*O*-Glc*p*	Roots (CHI) [14]
Monoterpene glycosides	
3,7-Dimethyl-8-(Glc*p*)-1,6-octadiene-3-ol (betulalbuside A)	Roots (CHI) [14]
Vervenone 10-*O*-Glc*p*	Roots (CHI) [16]
Norsesquiterpene glycosides	
(9*S*)-Drummondol-9-*O*-Glc*p*	Roots (CHI) [14]
Phenols	
Catechol	Roots (CHI) [17]
Benzoic acids	
Isovanillic acid	Roots (CHI) [17]
Benzyl glycosides	
Benzyl *O*-Glc*p*	Roots (CHI) [16]
3-Methoxy-4-hydroxy-propiophenone 4-*O*-Glc*p* (praeroside)	Roots (CHI) [18]
Tachioside	Roots (CHI) [18]
Isotachioside	Roots (CHI) [17]
Allyl benzenes	
2-Phenylethyl *O*-Glc*p*	Roots (CHI) [14]
4-Hydroxy-1-allylbenzene 3-*O*-(6″-*O*-Xyl*p*)-Glc*p*	Roots (CHI) [13]
Phenylpropanoids	
Tyrosol	Roots (CHI) [17]
Coniferin	Roots (CHI) [18]
Drupanin 4-*O*-(6″-*O*-Glc*p*)-Glc*p* (dissectumoside)	Roots (CHI) [17]
Ferulic acid	Roots (CHI) [16]
(*E*)-4-(3-Methoxy-prop-1-en-1-yl)-phenol	Roots (CHI) [16]
(*E*)-3-(4-Hydroxy-3-methoxyphenyl)-2-propenoic acid 2-(4-hydroxyphenyl) ethyl ester	Roots (CHI) [16]
Benzofurans	
6-Carboxylethyl-benzofuran 5-*O*-(2″-*O*-Xyl*p*)-Glc*p*	Roots (CHI) [14]
6-Methoxycarbonylethyl-benzofuran 5-*O*-(2″-*O*-Xyl*p*)-Glc*p*	Roots (CHI) [19]
Neolignans	
(7*S*,8*R*)-Dehydrodiconiferyl alcohol 4-*O*-Glc*p*	Roots (CHI) [13]
(7*S*,8*R*)-Dehydrodiconiferyl alcohol 4-*O*-Glc*p*-9′-*n*-butanol ether	Roots (CHI) [13]
(2*S*,3*S*,1′*S*,2′*R*)-2,3-Dihydro-5-(1′,2′-dihydroxypropyl)-2-(4-hydroxy-3-methoxyphenyl)-7-methoxy-3-methylbenzofuran	Roots (CHI) [13]
Simple coumarins	
Umbelliferone	Roots (TJK) [20]
	Herb (CHI) [21]
7-Isopentyloxycoumarin	Roots (MON) [22]
Scopoletin	Roots (CHI) [18]
	Herb (CHI) [21]
Isoscopoletin 6-*O*-Glc*p*	Roots (CHI) [23]
Isofraxidin 6-*O*-Glc*p* (eleutheroside B1)	Roots (CHI) [23]
Furanocoumarins linear	
Bergaptene	Roots (CHI) [13], (TJK) [20]
Isopimpinellin	Roots (CHI) [13], (TJK) [20], (MON) [22]
	Herb (CHI) [21]
Phellopterin	Herb (CHI) [21]
Byakangelicin	Herb (CHI) [21]
Xanthotoxin	Roots (CHI) [13]
Xanthotoxol	Roots (CHI) [18]
Imperatorin	Roots (CHI) [13]
Heraclenol	Roots (CHI) [15]
Heraclenol 3″-*O*-methyl ester	Roots (CHI) [16]
Heraclenol 3″-*O*-Glc*p*	Roots (CHI) [23]
Heraclenol 2″-*O*-Fer	Roots (CHI) [23]
Pabularinone	Roots (CHI) [15]
Isogosferol	Roots (CHI) [15]
Furanocoumarins linear dimeric	
Candinol C	Roots (CHI) [16]
Rivulobirin C	Roots (CHI) [16]
Rivulobirin D	Roots (CHI) [16]
Furanocoumarins angular	
Angelicin	Roots (CHI) [13], (TJK) [20]
Isobergaptene	Roots (CHI) [18], (MON) [22]
	Herb (CHI) [21]
Heramotol 6-*O*-Glc*p*	Roots (CHI) [18]
Sphondin	Roots (TJK) [20]
	Herb (CHI) [21]
Pimpinellin	Roots (TJK) [20], (MON) [22]
	Herb (CHI) [21]
Dihydrofuranocoumarins linear	
(9*R*,10*R*)-9,10-Dihydro-10-hydroxy-9-methoxy-bergapten (dissectumol)	Roots (CHI) [16]
Dihydrofuranocoumarins angular	
Apterin	Roots (CHI) [13]
Apterin 6″-*O*-Glc*p*	Roots (CHI) [19]
Hermandiol 5′-*O*-Glc*p* (yunngnoside B)	Roots (CHI) [15]
Dihydropyranocoumarins angular	
5,6-Dihydropyranocoumarin	Roots (CHI) [16]
Sterols	
β-Sitosterol	Herb (CHI) [21]
Daucosterol	Herb (CHI) [21]

^a^ Abbreviations: Fer―feruloyl; Frc*p*―fructopyranose; Glc*p*―glucopyranose; Xyl*p*―xylopyranose. ^b^ Origin: CHI―China; MON―Mongolia; TJK―Tajikistan.

**Table 2:** Regression equations, correlation coefficients (r^2^), standard deviation (S_YX_), limits of detection (LOD), limits of quantification (LOQ), linear ranges, relative standard deviations (RSD) for intra-day and inter-day precisions, and recovery of spiked samples (REC) for six reference standards.

Compound	a ^a^	b ^a^	Correlation Coefficient (r^2^)	S_YX_	LOD/LOQ (µg/mL)	Linear Range (µg/mL)	RSD% (Intra-Day)	RSD% (Inter-Day)	Recovery of Spiked Sample REC%
Heraclenin	3.4511	−0.9526	0.9896	0.36∙10^−2^	0.003/0.010	0–250	0.96	1.43	99.63
Oxypeucedanin	2.5481	−0.5231	0.9963	0.22∙10^−2^	0.002/0.009	0–250	0.99	1.28	100.70
Imperatorin	4.6210	−0.8694	0.9906	0.42∙10^−2^	0.003/0.009	0–250	1.03	1.52	98.94
Phellopterin	3.8637	−0.9005	0.9922	0.28∙10^−2^	0.002/0.007	0–250	0.97	1.11	99.52
Isoimperatorin	2.8631	−0.2634	0.9850	0.14∙10^−2^	0.002/0.005	0–250	1.06	1.27	100.93
Ostruthin	2.5387	−0.2622	0.9899	0.10∙10^−2^	0.001/0.004	0–250	0.99	1.14	100.52

^a^ Calibration equation parameter: y = a × x + b.

**Table 3:** Physico–chemical characteristics of *Heracleum dissectum* seed oil.

Parameter	Value
Yield (% dry seed weight)	10.52 ± 0.15
Viscosity (cP)	62.1 ± 1.2
Specific gravity (g/mL)	0.929 ± 0.018
Refractive index	1.472 ± 0.044
pH	6.20 ± 0.05
Peroxide value (mEq. peroxide/kg)	6.28 ± 0.18
Acid value (mg KOH/g)	0.52 ± 0.01
Saponification value (mg KOH/g)	173.82 ± 3.47
Iodine value (g of I_2_/100 g)	105.37 ± 2.10
Unsaponifiable matter (% *w*/*w*)	0.92 ± 0.02
Melting point (°C)	−25.3 ± −0.3
Chlorophyll a content (mg/L)	297.38 ± 5.94
Chlorophyll b content (mg/L)	66.70 ± 1.42
Carotenoid content (mg/L)	233.94 ± 4.67
Essential oil content (% *v*/*v*)	32.31 ± 0.62
Coumarin content (% *w*/*w*)	24.52 ± 0.51
Fatty acids (% of total FA content)	
Lauric acid (C12:0)	0.1 ± 0.0
Myristic acid (C14:0)	0.1 ± 0.0
Pentadecanoic acid (C15:0)	0.1 ± 0.0
Palmitic acid (C16:0)	5.2 ± 0.1
Palmitoleic acid (C16:1n7c)	0.1 ± 0.0
Heptadecanoic acid (C17:0)	0.1 ± 0.0
Stearic acid (C18:0)	1.2 ± 0.0
Petroselinic acid (C18:1n12c)	48.3 ± 0.9
Oleic acid (C18:1n9c)	10.2 ± 0.2
*cis*-Vaccenic acid (C18:1n7c)	0.8 ± 0.0
Linoleic acid (C18:2n6c)	28.3 ± 0.6
α-Linolenic acid (C18:3n3)	0.9 ± 0.0
Arachidic acid (C20:0)	0.4 ± 0.0
Behenic acid (C22:0)	0.1 ± 0.0
Lignoceric acid (C24:0)	0.1 ± 0.0

**Table 4:** Volatile components of *H. dissectum* seed oil.

Compound	RI ^a^	Content, %	Identification ^b^
Octanal	1003	1.2	i, ii, iii
Limonene	1027	0.4	i, ii, iii
Benzyl alcohol	1033	0.7	i, ii, iii
Octanol	1071	1.4	i, ii, iii
Hexyl butyrate	1192	3.0	i, ii
Decanal	1205	0.6	i, ii, iii
Octyl acetate	1214	67.8	i, ii, iii
Hexyl 2-methylbutyrate	1237	8.5	i, ii
Bornyl acetate	1286	0.1	i, ii, iii
Octyl isobutyrate	1345	1.4	i, ii
Octyl 2-methyl isobutyrate	1354	9.6	i, ii
Hexyl hexanoate	1387	0.5	i, ii, iii
Octyl butyrate	1391	0.6	i, ii
Decyl acetate	1411	0.2	i, ii, iii
Octyl 2-methylbutyrate	1437	0.9	i, ii
Germacrene D	1485	0.2	i, ii, iii
δ-Cadinene	1525	0.1	i, ii, iii
*E*-Nerolidol	1564	0.1	i, ii, iii
Octyl hexanoate	1585	1.1	i, ii, iii
Nonyl pentanoate	1588	0.1	i, ii
Octyl octanoate	1778	1.2	i, ii, iii
Total		99.7	

^a^ RI: Retention index determined on a HP-5 column relative to a series of *n*-alkanes (C_9_–C_29_). ^b^ Methods of identification: i, retention index; ii, mass spectrum; iii, co-injection with an authentic sample.

**Table 6:** Content of six coumarins in *H. dissectum* seed oil during storage, mg/mL ± S.D.

Storage Duration	Heraclenin	Oxypeucedanin	Imperatorin	Phellopterin	Isoimperatorin	Ostruthin	Total
Before storage (control samples)							
Sample 1	10.48 ± 0.21	3.23 ± 0.06	153.05 ± 3.06	37.12 ± 0.74	29.52 ± 0.59	5.02 ± 0.11	238.42
Sample 2	9.53 ± 0.19	1.18 ± 0.02	126.11 ± 2.53	42.10 ± 0.84	25.86 ± 0.52	4.59 ± 0.09	209.37
Sample 3	10.26 ± 0.21	5.76 ± 0.11	108.83 ± 2.19	30.83 ± 0.63	20.82 ± 0.40	4.64 ± 0.08	181.14
Sample 4	5.14 ± 0.11	0.93 ± 0.02	128.41 ± 2.57	32.63 ± 0.66	11.67 ± 0.23	5.09 ± 0.10	183.87
Room storage (20 °C; treated sample 1)							
1 year	9.43 ± 0.19 *	2.93 ± 0.04 *	140.81 ± 2.85 *	33.04 ± 0.67 *	26.56 ± 0.54 *	4.41 ± 0.08 *	217.18
2 years	8.91 ± 0.17 *	2.77 ± 0.04 *	128.52 ± 2.59 *	31.12 ± 0.61 *	24.21 ± 0.47 *	4.16 ± 0.07 *	199.69
3 years	8.17 ± 0.16 *	2.42 ± 0.03 *	110.19 ± 2.26 *	26.76 ± 0.52 *	20.66 ± 0.40 *	3.81 ± 0.05 *	172.01
Cold storage (1 °C; treated sample 1)							
1 year	10.37 ± 0.20	3.18 ± 0.06	149.99 ± 3.00	36.75 ± 0.74	29.20 ± 0.58	4.97 ± 0.09	234.46
2 years	10.15 ± 0.19	3.10 ± 0.06	148.45 ± 2.96	36.37 ± 0.71	28.38 ± 0.54	4.86 ± 0.09	231.31
3 years	9.85 ± 0.18 *	3.04 ± 0.06 *	143.87 ± 2.85 *	32.26 ± 0.63 *	27.75 ± 0.52 *	4.76 ± 0.08 *	221.53
Freeze storage (−20 °C; treated sample 1)							
1 year	10.45 ± 0.21	3.21 ± 0.06	152.80 ± 3.05	37.04 ± 0.73	29.06 ± 0.60	4.92 ± 0.11	237.48
2 years	10.37 ± 0.20	3.17 ± 0.06	151.48 ± 3.01	36.70 ± 0.72	29.14 ± 0.58	4.90 ± 0.11	235.79
3 years	10.16 ± 0.19	3.10 ± 0.06	149.90 ± 2.99	36.10 ± 0.70	28.82 ± 0.56	4.84 ± 0.11	232.92

Asterisk indicates a significant difference (*p* < 0.05) vs. before storage level.

**Table 7:** Effect of *H. dissectum* seed oil nanosuspension on the cerebral hemodynamics and the necrosis zone in rats with cerebral ischemia.

Parameter	Experimental Group (*n* = 15 For All Groups)					
	Sham-Operated Group	Negative Control	EGB761	HSO, 0.1 mL/kg	HSO, 0.5 mL/kg	HSO, 1 mL/kg
Cerebral Blood Flow, sm/sec	4.10 ± 0.25	1.25 ± 0.10 ^a^	2.63 ± 0.21 ^ab^	1.45 ± 0.11 ^ac^	2.28 ± 0.22 ^ab^	3.11 ± 0.26 ^abc^
Necrosis Zone Area, %	-	41.52 ± 3.73	21.60 ± 1.95 ^b^	42.62 ± 4.69 ^c^	30.38 ± 2.78 ^bc^	18.56 ± 1.69 ^bc^

Letters (^a–c^) indicates a significant difference (*p* < 0.05) vs. sham-operated animals’ group (^a^), negative control group (^b^), and EGB761 reference group (^c^).

## Data Availability

Not applicable.

## Supplementary Materials

The following supporting information can be downloaded at: https://www.mdpi.com/article/10.3390/life13051112/s1, Figure S1: Overlapped FTIR spectra of *H. dissectum* seed oil and impertorin, octyl acetate, and petroselinic acid; Table S1: Reference standards used for the qualitative and quantitative analysis by HPLC-DAD-ESI-tQ-MS assays.
