# Supplementary material for: Hogweed Seed Oil: Physico–Chemical Characterization, LC-MS Profile, and Neuroprotective Activity of Heracleum dissectum Nanosuspension
Source: Life (Basel). 2023 Apr 29;13(5):1112. doi: 10.3390/life13051112 (PMC10220559; doi:10.3390/life13051112)
Supplement: Supplementary file 1 [file life-13-01112-s001.zip › life-2369966-supplementary.pdf]

life

Article

Hogweed Seed Oil: Physico-Chemical Characterization, LC-MS Profile, and Neuroprotective Activity of *Heracleum dissectum* Nanosuspension

Daniil N. Olennikov [1,*] and Nadezhda K. Chirikova [2]

[1] Laboratory of Medical and Biological Research, Institute of General and Experimental Biology, Siberian Division, Russian Academy of Science, 6 Sakhyanovoy Street, 670047 Ulan-Ude, Russia
[2] Department of Biochemistry and Biotechnology, North-Eastern Federal University, 58 Belinsky Street, 677027 Yakutsk, Russia; hofnung@mail.ru
* Correspondence: olennikovdn@mail.ru; Tel.: +7-902-160-06-27

Figure S1. Overlapped FTIR spectra of *H. dissectum* seed oil (black) and impertorin (red), octyl acetate (green), and petroselinic acid (blue).

Table S1. Reference standards used for the qualitative and quantitative analysis by HPLC-DAD-ESI-tQ-MS assays.

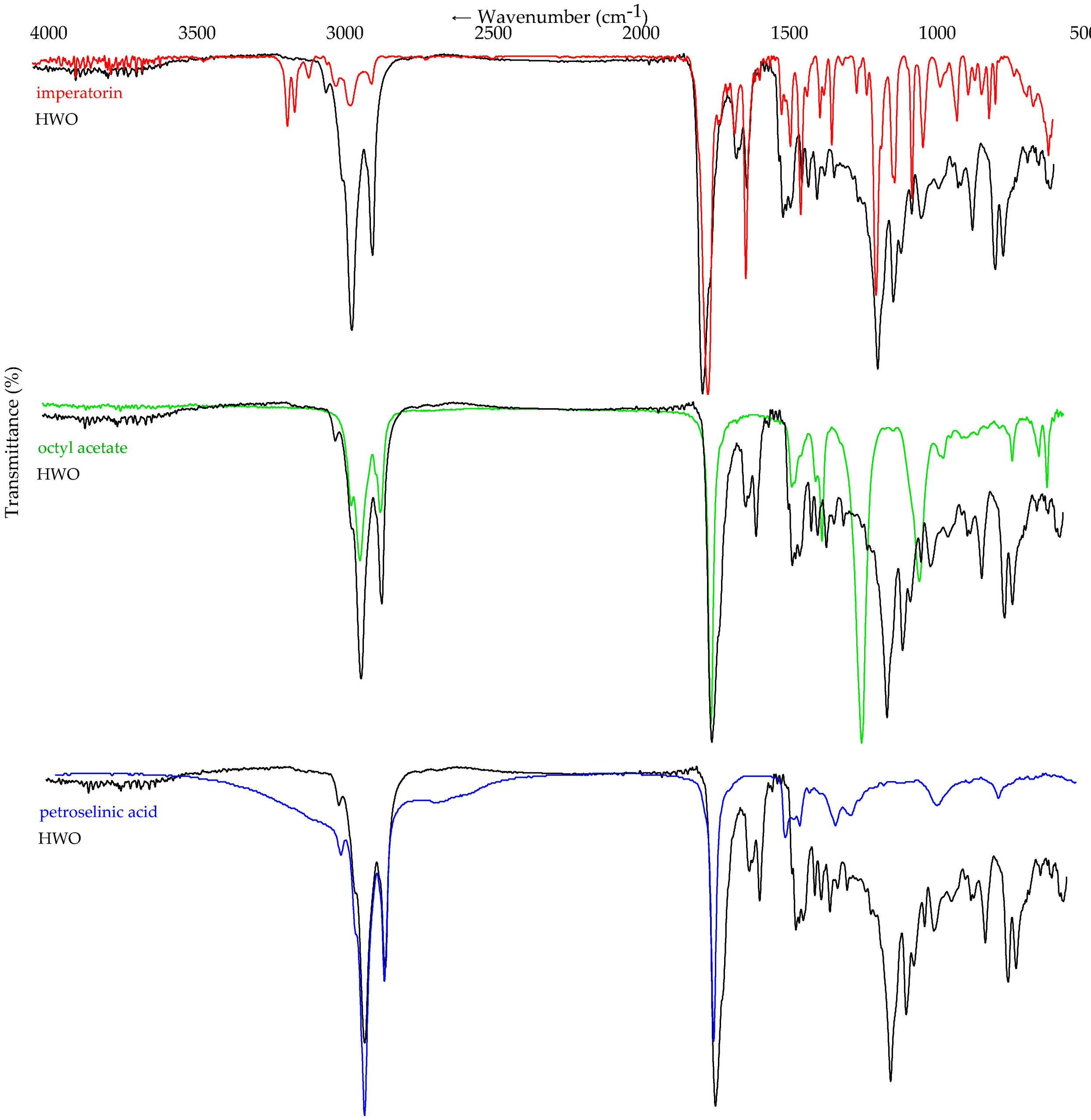

Figure S1. Overlapped FTIR spectra of *H. dissectum* seed oil (black) and impertorin (red), octyl acetate (green), and petroselinic acid (blue).

Table S1. Reference standards used for the qualitative and quantitative analysis by HPLC-DAD-ESI-tQ-MS assays.

No [a]	Compound	Standard [a]	Manufacturer (Cat. no) [b]	Purity, (≥) %
**1**	Isofraxetin	Fraxetin	Selleck (S9503)	99.85
**2**	Esculetin	Esculetin	Sigma (PHL80449)	95.00
**3**	Umbelliferone	Umbelliferone	Sigma (H24003)	99.00
**4**	Scopoletin	Scopoletin	Sigma (S2500)	99.00
**5**	Fraxetin	Fraxetin	Selleck (S9503)	99.85
**6**	Heraclenol	Heraclenol	Sigma (SMB00125)	95.00
**7**	Oxypeucedanin hydrate	Oxypeucedanin hydrate	MCE (HY-N2254)	99.94
**8**	Byakangelicin	Byakangelicin	AOBIOUS (APB0102)	98.00
**9**	Coumarin	Coumarin	Sigma (C4261)	99.00
**10**	Herniarin	Herniarin	MCE (HY-N1366)	99.92
**11**	Psoralene	Psoralene	Sigma (P8399)	99.00
**12**	Angelicin	Angelicin	MCE (HY-N0763)	99.86
**13**	Xanthotoxin	Xanthotoxin	Sigma (56448)	98.00
**14**	Bergapten	Bergapten	Sigma (69664)	99.00
**15**	Heraclenin	Heraclenin	BioCrick (BCN5187)	98.00
**16**	Pimpinellin	Pimpinellin	MCE (HY-N0438)	99.27
**17**	Byakangelicol	Byakangelicol	BioCrick (BCN5015)	98.00
**18**	Pranferol	Pranferol	BenchChem (B192152)	98.00
**19**	Alloimperatorin	Alloimperatorin	BioCrick (BCC8116)	98.00
**20**	Isooxypeucedanin	Isooxypeucedanin	AbMole (M18642)	98.00
**21**	Heracol	Byakangelicin	AOBIOUS (APB0102)	98.00
**22**	Oxypeucedanin	Oxypeucedanin	Sigma (PHL89876)	98.00
**23**	Oxypeucedanin isomer	Oxypeucedanin	Sigma (PHL89876)	98.00
**24**	Imperatorin	Imperatorin	MCE (HY-N0285)	98.00
**25**	Phellopterin	Phellopterin	BioCrick (BCN2637)	98.00
**26**	Isoimperatorin	Isoimperatorin	MCE (HY-N0286)	98.93
**27**	Cnidilin (isophellopterin)	Cnidilin (isophellopterin)	AOBIOUS (APB0656)	98.00
**28**	Farnesiferol C	Farnesiferol C	Sigma (SMB01056)	90.00
**29**	Cnidicin	Cnidicin	Sigma (50014)	95.00
**30**	Auraptene isomer	Auraptene	Sigma (A9861)	98.00
**31**	Bergamottin isomer	Bergamottin	Sigma (PHL89868)	95.00
**32**	8-Geranyloxypsoralen	8-Geranyloxypsoralen	Sigma (CDS010431)	95.00
**33**	Auraptene	Auraptene	Sigma (A9861)	98.00
**34**	Bergamottin	Bergamottin	Sigma (PHL89868)	95.00
**35**	Ostruthin	Ostruthin	Sigma (SMB00113)	95.00
**36**	5-Geranyl-7-methoxycoumarin	5-Geranyl-7-methoxycoumarin	Sigma (52006)	95.00
**38**	5-Geranyl-7-methoxycoumarin isomer	5-Geranyl-7-methoxycoumarin	Sigma (52006)	95.00

[a] Standards were used in qualitative ([A]) or/and quantitative analysis ([B]). [b] Manufacturers list: AbMole—AbMole BioScience (Houston, TX, USA); AOBIOUS—AOBIOUS Inc. (Gloucester, MA, USA); BenchChem—BenchChem (Austin, TX, USA); BioCrick—BioCrick (Chengdu, Sichuan, PRC); MCE—MCE Med Chem Express (Monmouth, NJ, USA); Sigma—Sigma-Aldrich (St. Louis, MO, USA); Selleck—Selleck Chemicals (Houston, TX, USA).
